# Effects of intimate partner violence and homophobic bullying on ART adherence among young Thai men who have sex with men: a causal mediation analysis

**DOI:** 10.1186/s12889-025-22055-1

**Published:** 2025-03-25

**Authors:** Doug H. Cheung, Alexis N. Reeves, Worawalan Waratworawan, Yamol Kongjareon, Thomas E. Guadamuz

**Affiliations:** 1 Mahidol Center for Health, Behavior and Society, Faculty of Tropical Medicine, Mahidol University, 420/6 Ratchawithi Road, Ratchathewi, Nakhon Pathom, Bangkok 10400 Thailand; 2 Jockey Club School of Public Health, Chinese University of Hong Kong, Hong Kong, China; 3 Department of Epidemiology and Population Health, Stanford University School of Medicine, Stanford, USA; 4 Department of Society and Health, Faculty of Social Sciences and Humanities, Mahidol University, Nakhon Pathom, Thailand; 5 John F. Kennedy School of Government, Harvard University, Cambridge, USA

**Keywords:** HIV/AIDS, Men who have sex with men, ART adherence, Victimization, Intimate partner violence, Thailand, Bullying, Depression, Longitudinal study, Causal mediation analysis

## Abstract

**Background:**

Adherence to antiretroviral therapy is crucial for determining health outcomes and secondary HIV transmission among people living with HIV/AIDS. Young men who have sex with men (YMSM) living with HIV are often challenged by the prevailing experiences of psychosocial stressors such as intimate partner violence and homophobic bullying, which may negatively affect their HIV care engagement.

**Methods:**

This study is the first to utilize a prospective cohort design (*N* = 185) involving YMSM living with HIV in Thailand. We examined the effects of intimate partner violence and homophobic bullying on ART adherence. We also tested the mediating effect of depression on the relationship between intimate partner violence and homophobic bullying and ART adherence.

**Results:**

We found that intimate partner violence (AOR: 2.58, 95% CI: 1.13, 5.42) and homophobic bullying (AOR: 2.40, 95% CI: 1.26, 4.48) were associated with subsequent ART nonadherence. Moreover, depression partially mediated 17.4% (95% CI: 0.75%–56%) of the effect of homophobic bullying on ART nonadherence.

**Conclusions:**

The results suggest that tailored interventions to optimize ART adherence should address the impact of intimate partner violence and homophobic bullying among YMSM living with HIV. Screening and subsequent treatment of depression alone may not be sufficient to address the effects of intimate partner violence, homophobic bullying, and possibly other MSM-specific psychosocial stressors on ART adherence.

## Introduction

Adherence to antiretroviral therapy (ART) is pivotal for establishing and sustaining viral load suppression and preventing disease progression and secondary transmission among people living with HIV. To meet UNAIDS 95–95-95 targets to end AIDS, 95% of people living with HIV should know their HIV status, 95% of those who know their HIV status should be on ART, and 95% of those on ART should be virally suppressed [1]. Since 2014, ART has been available free of charge through national health insurance in Thailand [2]. However, Thai men who have sex with men (MSM), a key population comprising 41% of all new infections, still have inadequate ART adherence and engagement in HIV care programs [3, 4]. National data from Thailand found that only 79.5% of MSM who started ART remained under treatment a year later. Only 47% of Thai MSM self-rated their ART adherence as “very good” or “excellent” in the past 3 months [3, 4]. Thus, significant public health efforts are needed to engage Thai MSM living with HIV [1].

Young Thai MSM (YMSM; aged 18–24 years old) are disproportionately impacted by the HIV epidemic in Thailand [5, 6]. With an elevated HIV incidence of 7.4 per 100 person-years between 2006 and 2014, YMSM is among the largest group of newly HIV-infected subpopulations in Thailand [5]. Young and adolescent MSM living with HIV are presented with unique challenges to HIV care engagement, including adverse childhood experiences, familial and peer violence, and victimization [7, 8]. More broadly, sexual and gender minorities experience heightened exposure to early life trauma and interpersonal violence, while YMSM could be especially vulnerable to these psychosocial stressors [9, 10]. In particular, exposure to traumatic experiences and interpersonal violence may have unique impacts on YMSM living with HIV [11]. Hirshfield et al. found that MSM in the US with past-year IPV were more likely to have suboptimal engagement in HIV care, and that those who reported past-year IPV were more likely to be younger (18–29 years old vs. 50 +) [12]. Furthermore, mental illness has been proposed as a potential mechanism by which IPV affects HIV-related health outcomes; however, it is unclear whether the treatment of mental illness can improve IPV-related disruption of ART adherence [13].

Recent evidence suggests that bullying experiences negatively affect ART adherence and other health-related outcomes among young PLWH in sub-Saharan Africa [14–16]. Homophobic bullying is another common form of trauma experienced by sexual minorities. It is associated with a range of adverse health consequences, such as depression and post-traumatic stress disorder (PTSD) [17, 18]. Despite being known as a “gay paradise” among international tourists and expats, youth and adolescents in Thailand endure pervasive homonegative social environments [19]. A study in 2014 found that more than half of LGBT-identified secondary school Thai students had been bullied; bullied students were more likely to be depressed and suicidal [20, 21]. YMSM living with HIV in Thailand may experience similar episodes of homophobic bullying, with negative mental health consequences that interrupt their ART adherence. However, research is yet to elucidate this relationship.

Worldwide evidence has shown that depression impedes ART adherence in PLWH [22]. Evidence suggests that the detection and subsequent treatment of depression, whether through psychological interventions or the use of antidepressants, improves ART adherence [23, 24]. MSM with HIV experience similarly elevated rates of depression; however, it is unclear whether treatment of depression would have a similar effect on ART adherence as seen in heterosexual persons living with HIV, considering the range of potentially unique psychosocial adversities experienced by MSM with HIV, such as IPV and homophobic bullying [25, 26]. To elucidate the mechanisms underlying these psychosocial adversities and ART adherence, the present study sought to investigate the effects of IPV and homophobic bullying on ART adherence using a unique dataset consisting of a prospective cohort of YMSM living with HIV in Bangkok. This is also the first prospective cohort study ever conducted involving YMSM living with HIV from Thailand. In addition, we examined whether depression mediated the effects of IPV and homophobic bullying on ART adherence among the study participants.

## Materials and methods

Data were drawn from a prospective cohort study of young MSM (aged 15–29 years old) living with HIV in Bangkok, Thailand collected from 2018 to 2019. We recruited 214 participants via community-based organization (CBO) partners, including the Poz Home Center Foundation and Rainbow Sky Association of Thailand (RSAT). Eligibility criteria included: (1) male sex at birth, (2) having had anal intercourse with a man in the past 6 months, (3) ability to speak Thai, (4) Thai nationality, (5) having lived in Bangkok for at least 6 months, (6) being between 15 and 29 years of age, (7) self-reporting of an HIV-positive status, and (8) consenting to be followed up online every four months for a total period of 12 months. Between January and February 2018, eligible participants were recruited via a web-based survey using Qualtrics software [27]. When participants completed the survey, they received 500 baht (17 USD) as compensation. Following the baseline survey, participants were asked to complete a follow-up survey at 4, 8, and 12 months. At the end of each survey administration, we asked participants whether they wanted to discuss personal concerns such as substance use and violence with a mental health professional. Upon receiving a positive response and subsequent consent, participants were referred to appropriate mental health professionals. Informed consent was obtained prior to enrollment. Mahidol University Institutional Review Board (IRB) reviewed and approved all study procedures (COA 2017/078.2803). This includes a waiver of parental permission for participants aged between 15 and 17 years. Mahidol University IRB granted this waiver on the grounds that more harm (e.g., unintended disclosure of sexual orientation or HIV status to parents or guardians) may occur among participants who are considered minors if parental permission is requested. A similar waiver of parental permission has been granted by Mahidol University IRB in previously published studies [28–31]. All participants between the ages of 15 and 17 years were subjected to the same online informed consent procedure as those aged 18 years and older. Data from this study are available via email with the corresponding author upon request.

### Measures

Participants self-reported the following sociodemographic characteristics: age (in years), employment status, educational level, average monthly income, sexual orientation, having a regular partner, and having received goods or opportunities (e.g., money, drugs and alcohol, mobile phones, mobile phone credits, clothes, bags, grades, or educational opportunities) in exchange for sex (i.e., engaged in sex work).

ART adherence was measured as the participant’s self-reported percentage of taking ART medications as prescribed over the past seven days, from 0 to 100% in 1% increments. Previous studies have shown that an 80–90% adherence level is adequate for viral suppression [32]. We categorized participants as ART non-adherent if they reported less than 90% adherence.

Depression was measured using the Center for Epidemiologic Studies Short Depression Scale (CESD-D-R), which assesses participants’ self-reported past 7 days of depression symptomology [33]. The scale consists of 10 items, and the total depression score is calculated by summing up all 10 items, with possible scores ranging from 0 to 30. A summed score equal to or greater than 10 indicates elevated depressive symptoms. Cronbach’s alpha coefficient for this scale at baseline was 0.759, indicating adequate internal consistency.

Intimate partner violence was assessed by asking the participants whether they had experienced the following experiences in the past six months (past four months in subsequent follow-up surveys): being hurt, hit, slapped in the body by a regular partner, casual sex partner, or male sex work partner; being forced to have sex by a regular partner or casual sex partner; being forced to have sex during sex work; being fondled; or having unwanted touching against their will. Participants who reported any of the above experiences were categorized as having experienced intimate partner violence.

Homophobic bullying was assessed by a single item asking whether the participants had ever been bullied (in the past four months in subsequent follow-up surveys) because of their sexual orientation or because they did not act like a boy or man.

### Data analysis

We conducted descriptive analyses using chi-square tests for categorical variables and two-sample t-tests for continuous variables to summarize and compare participant characteristics at baseline. First stratified by intimate partner violence in the past 6 months, then stratified by homophobic bullying. Since no missing values were reported among the baseline and subsequent follow-up surveys, we used a complete case analysis for all analyses.

Generalized linear mixed-effects models (GLMMs) were applied with the logit link function and unstructured covariance structure to estimate the prospective associations between sociodemographic and psychosocial variables (depression, intimate partner violence, and homophobic bullying) and ART non-adherence. The GLMM analysis examined the effects over time while accounting for the repeated measures within individuals, with the psychosocial variables and ART nonadherence included as time-varying in each wave. First, bivariate analyses were conducted between sociodemographic and psychosocial variables and ART nonadherence. We then estimated the adjusted parameters of the relationship between psychosocial variables (depression, intimate partner violence, and homophobic bullying) and ART nonadherence, adjusting for confounding effects within levels of age, education, income, regular partner status, and sex work. These confounding variables were identified a priori through a literature review. Psychosocial variables, such as depression, IPV, and homophobic bullying, were not adjusted for in the multivariable models because they are conceptualized as mediators between psychosocial variables and ART nonadherence [34].

We conducted causal mediation analyses of the mediating effects of depression on the relationship between intimate partner violence (or homophobic bullying) and ART nonadherence using the approach developed by Imai et al. [35]. In contrast to the GLMM analysis, which examined effects over time, a causal mediation analysis was conducted using the exposure (IPV or bullying), mediator (depression), and outcome (ART nonadherence) variables measured at the same time point, based on a counterfactual framework and the assumptions of the approach. This approach assumes that [1] the exposure is independent of all potential values of the outcome and mediating variables within levels of confounding variables and [2] the observed mediator is independent of all potential outcomes within the levels of confounding variables [35]. Therefore, unlike GLMMs that utilize time-varying variables across waves, lagged variables are not required for causal mediation analysis. The parameter estimates included the average direct effect (ADE), average causal mediation effect (ACME), total effect, and proportion mediated. ADE describes the association between intimate partner violence (or homophobic bullying) and ART nonadherence in a scenario where levels of exposure to depression (the mediator) are similar among participants exposed to intimate partner violence (or homophobic bullying). The ACME describes the elevated odds of ART nonadherence among participants exposed to intimate partner violence (or homophobic bullying) as mediated by depression. The mediated proportion was calculated as the ratio of the ACME to the total effect. This captures the importance of the mediator (i.e., % mediated via depression) in explaining the impact of exposure on the outcome. Sensitivity analyses were conducted to assess the impact of exposure-mediator interaction on causal mediation analyses and to assess the impact of ART nonadherence modeled as a continuous variable rather than a cutoff value of ≤ 90%.

All analyses were conducted using R software (version 4.3.1, R Foundation for Statistical Computing, Vienna, Austria). GLMMs were conducted using the “lme4” package in R [36], while causal mediation analyses were conducted using the “mediation” package in R [37]. Directed acyclic graphs were illustrated and analyzed using DAGGITY [38].

## Results

Of 214 eligible participants, 185 initiated ART at baseline and were included in the analysis. Three-fifths of the participants were aged 26 to 29 years, 75.1% had a tertiary education or above, 55.7% were employed full-time, 34.1% made 15,001 baht or above, and most of the participants (95.1%) were identified as gay. Nearly half (45.4%) of the participants reported having a regular partner and a quarter (25.4%) reported having ever provided sex work. Nearly half (43.2%) of the participants reported a clinically significant level of depressive symptoms at baseline, 11.9% reported being a victim of intimate partner violence at baseline, and 29.2% reported being subjected to homophobic bullying at baseline. Table 1 summarizes participant characteristics at baseline, stratified by the status of intimate partner violence from the past six months and ever experienced homophobic bullying at baseline. Compared to participants who had not experienced intimate partner violence in the past 6 months at baseline, participants who experienced intimate partner violence in the past 6 months at baseline were more likely to have an education level of secondary or below (21.7% vs. 8.6%, *p* = 0.02) and had symptoms of depression (17.5% vs. 7.6%, *p* = 0.04). Compared to participants who did not experience homophobic bullying, those who experienced homophobic bullying were more likely to be depressed (46.4% vs. 16.2%, *p* < 0.001). Table 2 contains the numbers and proportions of key variables over time (T1: baseline – T4: one year follow-up). Table 3 shows the bivariate analyses of sociodemographic variables and ART nonadherence. Participants with an income level between 5001 and 15,000 baht, compared to those with 15,001 baht or above (OR: 2.26, 1.22, 4.43, *p* = 0.01), were more likely to have ART nonadherence.

**Table 1:** Baseline characteristics of participants by intimate partner violence and homophobic bullying (*N* = 185)

Characteristic	Overall (*N* = 185)		Exposure to Intimate partner violence in the past 6 months (*n* = 22, 11.9%)			Exposure to Homophobic bullying (*n* = 54, 29.2%)		
	n	(%)	n	(%)	*P* value	n	(%)	*P* value
Age								
15–25	37	(20.0)	5	(13.5)	0.733	8	(21.6)	0.26
26–29	148	(80.0)	17	(11.5)		46	(31.1)	
Education								
Secondary or below	46	(24.9)	10	(21.7)	0.02	10	(21.7)	0.20
Tertiary or above	139	(75.1)	12	(8.6)		44	(31.7)	
Employment								
Full-time	103	(55.7)	12	(11.7)	0.53*	31	(30.1)	0.91
Part-time	38	(20.5)	3	(7.9)		10	(26.3)	
Unemployed or student	44	(23.8)	7	(15.9)		13	(29.5)	
Income								
5000 Bhat or below	35	(18.9)	4	(11.4)	0.76*	21	(33.3)	0.54
5001–15000 Bhat	87	(47.0)	9	(10.3)		22	(25.3)	
15001 Bhat or above	63	(34.1)	9	(14.3)		11	(31.4)	
Sexual Orientation								
Gay	176	(95.1)	21	(11.9)	0.94*	52	(29.5)	0.64*
Bisexual	8	(4.9)	1	(11.1)		2	(22.2)	
Having a regular partner								
Yes	84	(45.4)	11	(13.1)	0.65	21	(25.0)	0.25
No	101	(54.6)	11	(10.9)		33	(32.7)	
Ever provided sex work								
Yes	47	(25.4)	8	(17.0)	0.21	18	(38.3)	0.11
No	138	(74.6)	14	(10.1)		36	(26.1)	
Depression								
Yes	80	(43.2)	14	(17.5)	0.04	37	**(46.4)**	** < 0.001**
No	105	(56.8)	8	(7.6)		17	(16.2)	
ART nonadherence								
Yes	13	(7.0)	2	(15.4)	0.69	6	(46.2)	0.16
No	172	(93.0)	20	(11.6)		48	(27.9)	

Bolded = significant at *p* < 0.05

^*^Fisher exact test was used rather than X^2^

**Table 2:** Distributions of key variables from baseline to one-year follow up (T1-T4)

	T1	T2	T3	T4
ART Nonadherence, past 30 days				
Yes	13 (7.0)	14 (7.3)	22 (11.3)	12 (6.3)
No	172 (93.0)	177 (92.7)	173 (88.7)	179 (93.7)
Intimate partner violence, past 6 months				
Yes	22 (11.9)	19 (9.9)	11 (5.6)	11 (5.8)
No	163 (88.1)	172 (90.1)	184 (94.4)	180 (94.2)
Exposure to homophobic bullying, past 6 months				
Yes	54 (29.2)	28 (14.7)	21 (10.8)	16 (8.4)
No	131 (70.8)	163 (85.3)	174 (89.2)	175 (91.6)
Depression, past 2 weeks				
Yes	80 (43.2)	94 (49.2)	82 (42.1)	80 (41.9)
No	105 (56.8)	97 (50.8)	113 (57.9)	111 (58.1)

Follow-up time were spaced 3 months apart

**Table 3:** Bivariate associations of sociodemographic variable and ART nonadherence

	OR (95% CI)	*P* value
Age		
15–25	Ref	0.66
26–29	0.87 (0.48–1.68)	
Education		
Secondary or below	Ref	0.86
Tertiary or above	0.95 (0.52–1.82)	
Employment		
Full-time		
Part-time	1.38 (0.68–2.65)	0.35
Unemployed or student	1.34 (0.70–2.47)	0.36
Income		
5000 Bhat or below	1.43 (0.58–3.36)	0.42
5001–15000 Bhat	**2.26 (1.22–4.43)**	**0.01**
15,001 Bhat or above	Ref	
Sexual Orientation		
Gay	2.31 (0.47–41.63)	0.42
Bisexual	Ref	
Having a regular partner		
Yes	1.37 (0.81–2.33)	0.24
No	Ref	
Ever provided sex work		
Yes	1.67 (0.84–3.14)	0.12
No		

Bolded = significant at *p* < 0.05

In our bivariate analyses (Table 4), we found that depression (OR: 2.07, 95% CI: 1.22, 3.59, *p* < 0.001), intimate partner violence (OR: 2.41, 95% CI: 1.10, 4.88, *p* = 0.02), and homophobic bullying (OR: 2.32, 95% CI: 1.23, 4.22, *p* < 0.001) were significantly associated with ART nonadherence. After adjusting for age, education, income, regular partner status, and sex work, depression (AOR: 2.29, 95% CI: 1.16, 4.65, *p* = 0.01), intimate partner violence (AOR: 2.58, 95% CI: 1.13, 5.42, *p* = 0.02), and homophobic bullying (AOR: 2.40, 95% CI: 1.26, 4.48, *p* = 0.006) were significantly associated with ART nonadherence.

**Table 4:** Associations between psychosocial variables and ART nonadherence

	Unadjusted		Adjusted^a^	
	OR (95% CI)	*P*	AOR (95% CI)	*P*
Depression				
Yes	**2.07 (1.22–3.59)**	** < 0.001**	**2.29 (1.16–4.65)**	**0.01**
No	Ref		Ref	
Intimate Partner Violence				
Yes	**2.41 (1.10–4.88)**	**0.02**	**2.58 (1.13–5.42)**	**0.02**
No	Ref		Ref	
Homophobic bullying				
Yes	**2.32 (1.23–4.22)**	** < 0.001**	**2.40 (1.26–4.48)**	**0.006**
No	Ref		Ref	

Bolded = significant at *p* < 0.05

^a^Models adjusted for age, education, income, regular partner status and ever provided sex work

**Fig. 1:**
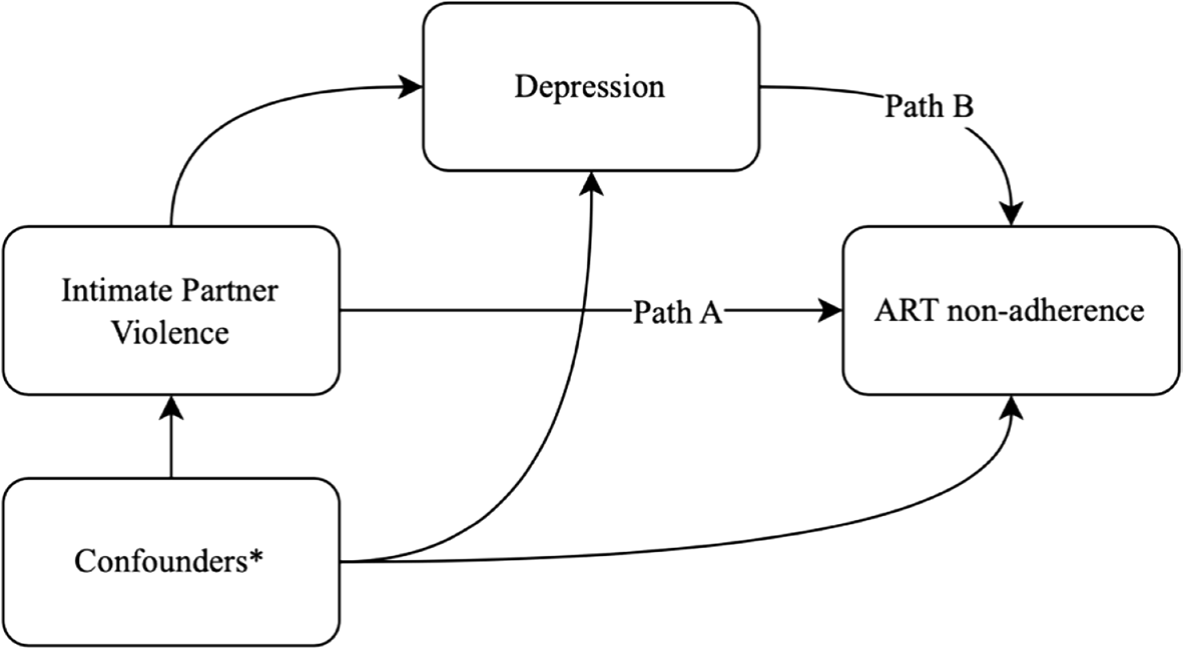
Directed acyclic graph (DAG) of the relationships between confounders, intimate partner violence, depression and ART nonadherence. *Confounding adjustment include age, education, income, and ever provided sex work. The full DAG is viewable at the following URL: https://dagitty.net/dags.html?id=eT9xZZVs

**Fig. 2:**
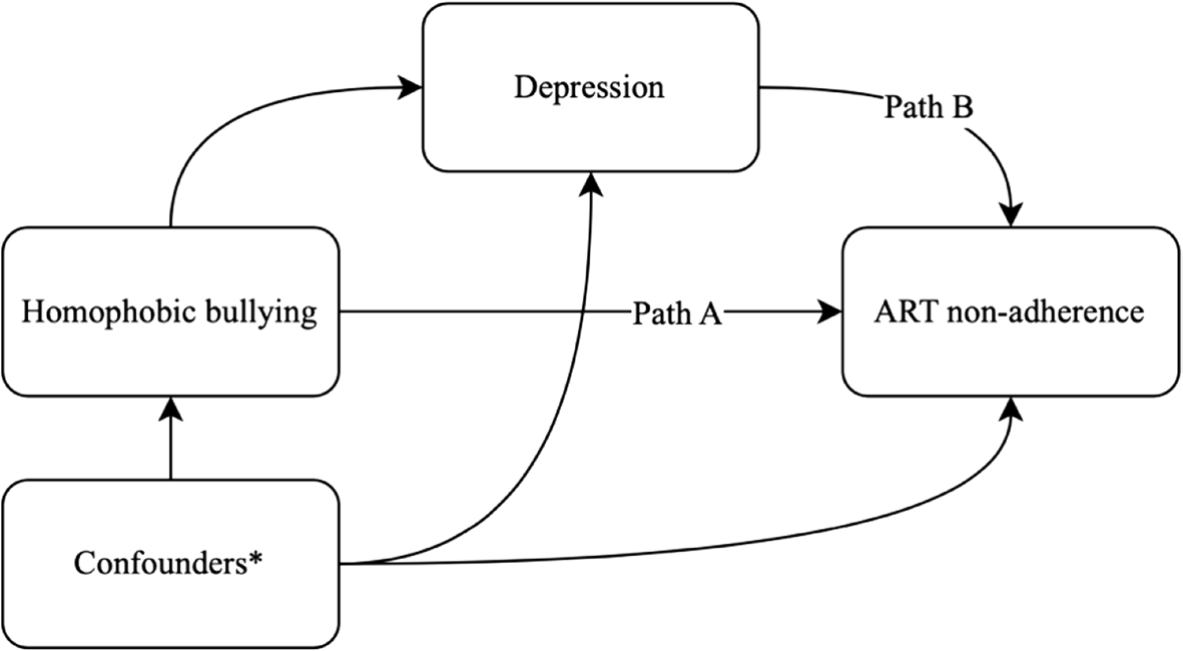
Directed acyclic graph (DAG) of the relationships between confounders, homophobic bullying, depression and ART nonadherence. *Confounding adjustment include age, education, income, and ever provided sex work. The full DAG is viewable at the following URL: https://dagitty.net/dags.html?id=eT9xZZVs

Table 5 presents parameter estimates from causal mediation analyses estimating the direct and indirect effects of intimate partner violence on ART nonadherence via depression and homophobic bullying on ART nonadherence via depression. In the first causal mediation analysis investigating the mediating role of depression on the relationship between intimate partner violence and ART nonadherence, intimate partner violence was positively associated with depression in the first step of the mediation model (AOR: 1.83, 95% CI: 1.11, 3.06, *p* = 0.02). At the same time, depression was positively associated with ART nonadherence (AOR: 1.96, 95% CI: 1.13, 3.46, *p* = 0.02). This resulted in an estimated average direct effect (ADE) of AOR:1.09 (95% CI: 1.01,1.22); however, we did not find evidence of an indirect effect since the average causal mediation effect (ACME) was not statistically significant (AOR: 1.01, 95% CI: 0.99, 1.02, *p* = 0.09). In the second causal mediation analysis investigating the mediating role of depression on the relationship between homophobic bullying and ART nonadherence, homophobic bullying was positively associated with depression in the first step of the mediation model (AOR: 3.17, 95% CI: 2.09, 4.93, *p* < 0.001). Meanwhile, depression was positively associated with ART nonadherence (AOR: 1.80, 95% CI: 1.02, 3.21, *p* = 0.02). This resulted in an estimated ADE of AOR:1.07 (95% CI: 1.01, 1.15, *p* = 0.03) and ACME of AOR:1.01 (95% CI: 1.00, 1.03, *p* = 0.03), suggesting that 17.4% (95% CI: 0.75%, 56%, *p* = 0.04) of the effect of homophobic bullying on ART nonadherence was mediated by depression. The relationships between confounders (age, education, income, regular partner status, ever-provided sex work), exposures (intimate partner violence and homophobic bullying), mediators (depression), and outcome (ART nonadherence) were conceptualized by directed acyclic graphs (DAGs), as illustrated in Figs. 1 and 2, respectively, to elucidate the minimum sufficient set of variables for confounding adjustments to estimate the total effects between exposures and outcome.

**Table 5:** Causal mediation analyses of intimate partner violence and homophobic bullying on ART nonadherence via depression

	AOR^a^	95% CI	*P* value
Intimate partner violence			
Intimate partner violence → Depression	**1.83**	**1.11, 3.06**	**0.02**
Depression → ART nonadherence	**1.96**	**1.13, 3.46**	**0.02**
Total effect	**1.10**	**1.02, 1.22**	**0.01**
Direct effect (not through depression, path A)	**1.09**	**1.01, 1.22**	**0.02**
Average causal mediation effect (through depression, path B)	1.01	0.99, 1.02	0.09
% Mediated	8.1%	−1.5%, 39%	0.10
Homophobic bullying			
Homophobic bullying → Depression	**3.17**	**2.09, 4.93**	** < 0.001**
Depression → ART nonadherence	**1.80**	**1.02, 3.21**	**0.02**
Total effect	**1.09**	**1.02, 1.17**	**0.008**
Direct effect (not through depression, path A)	**1.07**	**1.01, 1.15**	**0.03**
Average causal mediation effect (through depression, path B)	**1.01**	**1.00, 1.03**	**0.03**
% Mediated	**17.4%**	**0.75%, 56%**	**0.04**

Bolded = significant at *p* < 0.05

^a^Models adjusted for age, education, income, regular partner status and ever provided sex work

In the sensitivity analyses to test for exposure mediator interaction, there was no significant interaction between IPV and depression on ART nonadherence or homophobic bullying and depression on ART nonadherence (Tables 6 and 7). When ART nonadherence was modeled as a continuous variable, the mediation effect (ACME) did not remain significant, but the direction and magnitude of effect estimates remained in the hypothesized manner, resembling the main analysis results in Table 5.

**Table 6:** Sensitivity analyses assessing the impact of exposure-mediator interaction on casual mediation analyses’ parameter estimates

	AOR^a^	95% CI	*P* value
Intimate partner violence			
Intimate partner violence → Depression	**1.83**	**1.11, 3.06**	**0.02**
Depression → ART nonadherence	**1.91**	**1.05, 3.54**	**0.04**
Depression * Intimate partner violence → ART nonadherence	1.20	0.25, 6.80	0.83
Total effect	**1.11**	**1.02, 1.22**	**0.01**
Direct effect (not through depression, path A)	**1.10**	**1.01, 1.22**	**0.03**
Average causal mediation effect (through depression, path B)	1.01	0.99, 1.03	0.23
% Mediated	**7.1%**	**−4%, 38%**	**0.24**
Homophobic bullying			
Homophobic bullying → Depression	**3.17**	**2.09, 4.93**	** < 0.001**
Depression → ART nonadherence	1.82	0.96, 3.50	0.07
Depression * homophobic bullying → ART nonadherence	0.93	0.25, 4.07	0.92
Total effect	**1.08**	**1.02, 1.16**	**0.006**
Direct effect (not through depression, path A)	**1.07**	**1.01, 1.16**	**0.02**
Average causal mediation effect (through depression, path B)	1.01	0.99, 1.03	0.21
% Mediated	15.6%	−11.9%, 59%	0.21

Bolded = significant at *p* < 0.05

^a^Models adjusted for age, education, income, regular partner status and ever provided sex work

**Table 7:** Sensitivity analyses assessing the impact of ART adherence as a continuous variable on casual mediation analyses’ parameter estimates

	AORa	95% CI	*P* value
Intimate partner violence			
Intimate partner violence → Depression	1.65	0.98, 2.78	0.058
Depression → ART nonadherence	1.75	−0.14, −3.64	0.070
Intimate partner violence → ART nonadherence	1.68	−1.73, 5.10	0.33
Total effect	2.03	−1.46, 5.21	0.26
Direct effect (not through depression, path A)	1.81	−1.69, 4.93	0.32
Average causal mediation effect (through depression, path B)	0.22	−0.03, 0.64	0.11
% Mediated	7.4%	−0.87%, 1,16%	0.24
Homophobic bullying			
Homophobic bullying → Depression	**3.25**	**2.13, 4.96**	** < 0.001**
Depression → ART nonadherence	1.30	−0.63, 3.11	0.19
Homophobic bullying → ART nonadherence	**3.46**	**0.80, 6.11**	**0.011**
Total effect	**3.79**	**1.42, 6.39**	**0.002**
Direct effect (not through depression, path A)	**3.44**	**0.94, 6.08**	**0.004**
Average causal mediation effect (through depression, path B)	0.36	−0.14, 0.95	0.21
% Mediated	9.7%	−4.4%, 37%	0.18

Bolded = significant at *p* < 0.05

^a^Models adjusted for age, education, income, regular partner status and ever provided sex work

## Discussion

In this prospective cohort study, we evaluated the longitudinal association between intimate partner violence, homophobic bullying, and ART nonadherence using a novel community study of young MSM living with HIV in Bangkok. Our results suggest that experiencing intimate partner violence and homophobic bullying is associated with ART nonadherence. Moreover, we found that depression only partially mediated (17.4%, 95% CI: 0.75%, 56%) the relationship between homophobic bullying and ART adherence, whereas depression did not play a role in the relationship between IPV and ART adherence. Our results imply that potential interventions for treating depression alone may not adequately address the effects of IPV and homophobic bullying on HIV-related health outcomes among young Thai MSM living with HIV. These results suggest that tailored interventions to improve HIV-related outcomes should address the multifaceted forms of victimization experienced by young MSM living with HIV.

Among our cohort of young MSM living with HIV in Bangkok, those who experienced IPV were more likely to have suboptimal adherence to their ART regimens. This result is consistent with global evidence on the effect of IPV on ART adherence among women living with HIV [39]. Wang et al. found that among MSM living with HIV in China, the verbal form of IPV is associated with a lower CD4 + cell count [40]. In addition, the relationship between IPV and lower CD4 + cell counts is fully mediated by depression. In contrast, we found that depression did not play a significant mediating role in the relationship between IPV and ART adherence. This discrepancy could be because Wang et al.’s study examined the relationship with the verbal form of IPV, whereas our measures captured only physical and sexual forms of IPV. Depression may have different mediating roles in the different forms of IPV.

Additionally, depression may play a more vital role in determining the immunologic response (CD4 + cell count) than behaviors such as ART adherence. A decrease in CD4 + cell count may account for an additional immunological impact of depression, independent of ART adherence, which may explain the discrepancy in results across studies [41]. Our findings support a direct relationship between IPV and adherence to ART. MSM living with HIV who are experiencing IPV may have diminished self-efficacy in prioritizing their health and self-care, as observed among heterosexual women living with HIV [39].

Among our participants, those who experienced homophobic bullying were more likely to have suboptimal adherence to their ART regimens. Although literature on this relationship is scarce for MSM living with HIV, our results are consistent with those of studies investigating the relationship between victimization and HIV-related outcomes among adolescents in sub-Saharan Africa [14, 42]. Casale et al. found that psychological distress mediated the effect of bullying on ART adherence among adolescents living with HIV in South Africa [14]. However, we found that only a relatively small fraction of the effect of homophobic bullying on ART adherence was mediated by depression. The effect of homophobic bullying on HIV-related outcomes can likely be mediated through other psychosocial factors such as anxiety disorder, PTSD, and substance use [26]. Harkness et al. found that childhood sexual abuse, PTSD, anxiety disorder, depression, alcohol abuse, and polysubstance/stimulant use additively contributed to ART nonadherence among MSM [26]. These syndemic psychosocial adversities often act in consortia in affect various health outcomes of MSM [26, 43, 44]. To interrupt the negative health impacts of these syndemic sequelae, our study highlights the importance of developing and implementing tailored psychosocial interventions for MSM living with HIV who have a history of being bullied, both to improve mental well-being and optimize HIV-related outcomes.

In our previous study, which entailed the baseline data of this prospective cohort study, social support was negatively associated with ART nonadherence for those with increased depressive symptoms, suggesting the protective role of social support by interrupting the effect of depressive symptoms on ART nonadherence [45]. Social support may also play a protective role in the relationships between IPV, homophobic bullying, and ART nonadherence. However, we cannot provide such analyses because social support was only measured at baseline. A recent systematic review and meta-analysis found that peer support with routine medical care is superior to routine clinic follow-up in improving outcomes for people living with HIV, suggesting the potential role of peer-targeted tailored community interventions in improving ART adherence and other clinical outcomes for MSM living with HIV [46]. Future studies should investigate the protective roles of social support between a range of MSM-specific psychosocial stressors and exposures, such as internalized homophobia, discrimination, HIV stigma, early life trauma, substance and alcohol use, and HIV-related outcomes, including ART adherence.

The findings of this study should be interpreted with caution in light of these limitations. First, we recruited participants through CBO referrals, which may not be representative of the underlying population of YMSM living with HIV in Thailand. Those engaged with CBOs were more likely to have fewer victimization experiences, better ART adherence, and higher social and peer support, which may underestimate the relationship between our exposures and outcomes. Second, we used relatively simple measures of IPV and homophobic bullying, which may not adequately capture the full range of experiences of IPV and homophobic bullying. Such misclassifications may underestimate the relationship between exposure and outcomes. Third, unobserved confounding factors might be present in the relationship between the mediator and outcome; for example, personality factors could be associated with depression and ART adherence [47]. Such unmeasured confounding factors may violate the assumption of causal mediation analysis [35]. Nevertheless, many critical confounding variables that may approximate some of these characteristics were measured and controlled for in this study. In our sensitivity analysis, after the inclusion of the exposure-mediator interaction in the relationship between homophobic bullying, depression, and ART nonadherence, the average causal mediation effect was not statistically significant. This is probably due to a lack of power from our limited sample size; however, since the exposure-mediator interaction was insignificant, we did not include it in our final analysis. Similarly, we did not observe a significant mediation effect in the sensitivity analyses that modeled ART nonadherence as a continuous variable. Unfortunately, statistical significance was not found likely due to the limited variability in ART nonadherence scores and the relatively small sample size, which may have reduced the power to detect significant associations. Nevertheless, the direction and magnitude of the effects remained consistent with our primary analysis using the 90% adherence cutoff, suggesting that our findings are non-trivial to different specifications of the ART adherence variable.

Of note, only two participants were exposed to intimate partner violence and reported ART nonadherence at baseline. This sparsity in the data may have led to a nonstructural violation of the positivity assumption, resulting in unstable or inaccurate estimates [48]. Finally, we did not adjust for possible observed or unobserved time-varying confounders. Potential time-varying confounders include prior depression levels and prior ART adherence, which could bias the associations in the positive direction. Future research should consider g-computation and instrumental variable approaches to account for time-varying confounding.

## Conclusion

Our study is novel in its contribution to the literature by using the first prospective cohort study involving YMSM living with HIV conducted in Thailand. We found that experiences of IPV and homophobic bullying impede ART adherence, and that depression plays a relatively small mediating role in the relationship between homophobic bullying and ART adherence. In Thailand, HIV care facilities are beginning to integrate mental health screening and treatment for persons living with HIV [49]. Although the integration of mental health services into HIV care facilities is an essential first step, these programs can benefit from care providers who are competent to address MSM-specific psychosocial issues and their complex needs; therefore, proper screening, treatment, and care plans can be devised to provide optimal HIV care for MSM living with HIV [49, 50]. Our results motivate tailored interventions addressing the impacts of victimization on YMSM living with HIV and highlight the importance of MSM-competent integrated HIV care to holistically improve their HIV care continuum and mental health comorbidities.

## Data Availability

No datasets were generated or analysed during the current study.

## Abbreviations

YMSM: Young men who have sex with men
UNAIDS: Joint United Nations Programme on HIV/AIDS
HIV: Human immunodeficiency virus
PLWH: Persons living with human immunodeficiency virus
CBO: Community-based organization
ART: Antiretroviral therapy
IPV: Intimate partner violence
PTSD: Posttraumatic stress disorder
CESD-D-R: Center for Epidemiologic Studies Short Depression Scale
ACME: Average causal mediation effect
ADE: Average direct effect
OR: Odds ratio
AOR: Adjusted odds ratio
CI: Confidence interval
GLMMs: Generalized linear mixed effects models
